# “… give me a lawyer, dawg”: recognizing *youthful pleas* for advice and support

**DOI:** 10.3389/fpsyg.2024.1414305

**Published:** 2024-10-09

**Authors:** Miko M. Wilford, Annabelle Frazier

**Affiliations:** ^1^Department of Psychology, Iowa State University, Ames, IA, United States; ^2^School of Criminal Justice, Forensic Science and Security, University of Southern Mississippi, Hattiesburg, MS, United States

**Keywords:** legal decision making, guilty pleas, false guilty pleas, adjudication, juvenile crime

## Abstract

(1) Youth are particularly vulnerable to making suboptimal legal decisions due to added challenges in comprehension, as well as their ongoing neurological development.

(2) This fact is especially problematic in our *system of pleas,* which frequently expects defendants to determine the outcome of their own cases (by accepting or rejecting plea offers).

(3) To better ensure that guilty pleas entered by youthful defendants are indeed knowing, intelligent, and voluntary, legal counsel should be mandatory during any and all juvenile criminal proceedings.

(4) Further, an additional “translator” (similar to a *guardian ad litem*), who has specialized training in developmental psychology, should be present to effectively counsel and support youth defendants who might otherwise fail to comprehend the advice of their attorneys (and other court actors).

## Introduction

In Miranda v. Arizona (1966), the Supreme Court ruled that an interrogation must end if the suspect invokes his right to counsel. Subsequent decisions have required that this invocation be “unequivocal” and “unambiguous,” or the interrogation may continue (State v. Payne, 2002). At 22, Mr. Demesme twice waived his *Miranda* rights and submitted to police questioning. After repeated accusations and denials, he exclaimed, “… I know that I did not do it so why don’t you just give me a lawyer dawg cause this is not what’s up.” Yet the interrogation continued, and Demesme ultimately made self-incriminating statements. Demesme’s lawyer argued that his statement served as an invocation of counsel, but the Louisiana Supreme Court disagreed. Justices opined that Demesme’s “… ambiguous and equivocal reference to a ‘lawyer dog’ does not constitute an invocation of counsel that warrants termination of the interview …” and that detectives *reasonably* assumed he requested a four-legged animal that had passed the state Bar—a request they had no duty to fulfill (State v. Demesme, 2017). This interpretation spurred a barrage of criticism from the media and public, who saw no ambiguity in Demesme’s request for counsel even if coupled with the vernacular term, “dawg” (Stern, 2017).

In Fare v. Michael C (1979) the Supreme Court laid the groundwork for this decision by setting a high bar when evaluating the voluntariness of youths’ admissions of guilt. In this case, 16-year-old Michael C. was advised of his *Miranda* rights and asked if he wanted an attorney. Because of his long history with the juvenile court, Michael requested to speak to his probation officer instead. Having been told that the probation officer was unavailable, Michael proceeded to make incriminating statements. Michael’s attorney subsequently argued that a juvenile’s request to speak with any trusted adult should constitute an invocation of his Fifth Amendment right, just as if he had requested counsel. The Supreme Court disagreed, leaving individual courts to interpret each instance of interrogation. Writing for the majority, Justice Blackmun opined that,

*“…A juvenile's request to speak with his probation officer does not constitute a per se request to remain silent, nor is it tantamount to a request for an attorney…”*

In this policy brief, we contrast legal precedent pertaining to youths’ admissions of guilt, with research evidence surrounding youth decision-making, highlighting discrepancies between young people’s abilities and the legal standards to which they are held. We conclude by proposing practical strategies that may be used to protect youths in these, and related contexts.

### Protecting youthful defendants

Developmental research demonstrates the myriad ways in which youth may be negatively affected by their own cognitive and neurobiological immaturity (e.g., Cauffman and Steinberg, 2000). Current scientific consensus indicates that the human prefrontal cortex (responsible for reasoning, decision-making, and impulse control) does not reach full size and functionality until people are in their mid-twenties (Du, 2020). All the while, emotion processing systems (i.e., the limbic system) reach their full capacity earlier, making teens particularly sensitive to peer rejection and high-emotion situations (Silvers et al., 2012). This neurocognitive immaturity, coupled with heightened emotional reactivity, explains not only many youths’ acts of misconduct, but also their difficulties in navigating the legal system. Indeed, juvenile defense counsel routinely report that their clients are incompetent to make guilty plea decisions (Nemoyer et al., 2018), because they seem unable to fully understand the process, its consequences, and their rights (Zottoli and Daftary-Kapur, 2019).

In a series of Supreme Court decisions (Roper v. Simmons, 2005; Graham v. Florida, 2010; Miller v. Alabama, 2012; Montgomery v. Louisiana, 2016), judges recognized the significance of this research. The Supreme Court has limited the possibility of youth to receive sentences as severe as life-without-parole (Graham v. Florida, 2010), and several states banned life sentences for defendants under 18. In *Graham*, Justice Kennedy quoted the *Roper* decision,

“*Developments in psychology and brain science continue to show fundamental differences between juvenile and adult minds. For example, parts of the brain involved in behavior control continue to mature through late adolescence… Juveniles’ lack of maturity and underdeveloped sense of responsibility often result in impetuous and ill-considered actions and decisions.”*

### Understanding youthful pleas

Interestingly, Courts have recognized the role of impetuousness in youth criminal behaviors; yet the same vulnerabilities are unrecognized when evaluating the voluntariness of youthful statements. This is particularly problematic considering the frequency with which criminal convictions are decided, not by a jury verdict, but by a criminal defendant’s decision to plead guilty (Wilford and Bornstein, 2023). Because youthful defendants are regularly made to determine the outcome of their own cases (National Center for State Courts, 2023), the legal system must take steps to understand youthful vulnerabilities in this context.

Several of these vulnerabilities feature prominently in Joe Buffey’s wrongful conviction. In 2002, Buffey (then 19-years-old) falsely pled guilty to raping and robbing an 83-year-old woman, serving 15 years of a life sentence before he was exonerated. Buffey’s ordeal began when he waived his *Miranda* rights and made a false confession. Though he recanted immediately, he was encouraged to plead guilty before receiving discovery evidence that could have led to a dismissal. Buffey’s defense attorney exacerbated the pressure to plead guilty by emphasizing the threat of a long sentence. Even after DNA evidence cleared Buffey, he was pressured into pleading guilty again, in a “deal” that allowed him to maintain his innocence (North Carolina v. Alford, 1970) while avoiding re-trial.

Notably, both of Buffey’s plea decisions involved overweighting the short-term benefits of a certain, predictable, and speedy resolution, and underweighting the long-term direct and collateral consequences of the convictions these pleas carried. Indeed, youths’ decisions often overweight proximal (immediate) factors, while underweighting distal (delayed) consequences (e.g., Fountain and Woolard, 2018). Youth also underweight important variables when making legal decisions. In a sample of juvenile defendants, younger defendants (11–14-year-olds) were less impacted by strength of evidence when making plea decisions relative to older defendants (15–17-year-olds; Viljoen et al., 2005). Youths are also not immune to extralegal biases. Race play a significant role in plea outcomes among youthful offenders (Schissel, 1993; Tepfer et al., 2009), with minorities sometimes receiving relatively worse offers (Kutateladze et al., 2016), or being at a generally greater risk of pleading guilty falsely (Haney-Caron and Fountain, 2021). Youth are also more likely to accept plea offers when innocent (Helm et al., 2018; Redlich et al., 2019; Wilford and Khairalla, 2019). Many of these, and related risk factors, played significant roles in numerous wrongful convictions, including the now-infamous case of the Central Park Five (Norris and Redlich, 2013).

Younger children and teens are also more likely to comply with perceived authority figures, which further corresponds to increased guilty pleas (Grisso et al., 2003), even when innocent (Redlich and Shteynberg, 2016; Tepfer et al., 2009). Similar vulnerabilities also increase youths’ likelihood of making incriminating interrogation statements (August and Henderson, 2021), including false confessions (Tepfer et al., 2009). Once an incriminating statement has been recorded, the likelihood of pleading guilty increases (DiFava et al., 2023; Redlich, 2010; Scherr et al., 2020). In interviews of incarcerated youths, nearly 20% claimed to have pleaded guilty falsely, and many cited pressures from attorneys as a contributing factor (Malloy et al., 2014). Juvenile defendants’ plea decisions are clearly impacted by different factors than adults (Peterson-Badali and Abramovitch, 1993; Redlich and Shteynberg, 2016; Viljoen et al., 2005), with multiple differences for which scientific consensus is broad.

## Policy options and implications

In the United States, most youth defendants (99% by some estimates) are adjudicated in juvenile courts: specialty courts that follow civil procedure (Hockenberry, 2021). Intended to act as rehabilitative intervention agents for youth, officers of these courts are afforded significant discretion in determining the trajectory and outcomes of cases (Skowyra and Cocozza, 2007). For instance, some jurisdictions do not consider delinquency proceedings inherently adversarial, and therefore, do not *universally* provide counsel for defendants facing juvenile delinquency hearings (*In re: Gault*, 1967; Office of Juvenile Justice and Delinquency Prevention, 2018). Likewise, judges and prosecutors have many options in intervening with youths who enter the system. Formal dispositions in juvenile cases may vary the duration of institutional commitments (like adult prison sentences), but may also include “alternative” punishments, which can be qualitatively different. A wide variety of informal and diversionary dispositions are also common, and these too may be negotiated as part of a plea.

Furthermore, a small number of youths accused of violent crimes are “waived” or transferred to adult criminal courts, which broadens potential sentencing severity, up to and including a life sentence (Miller v. Alabama, 2012; but not the death penalty, see Roper v. Simmons, 2005). Thus, juvenile plea bargaining may include negotiations to avoid transfer to criminal court, and several other potential punitive and rehabilitative measures. Yet, because juvenile court hearings and records are not public, and because both formal and informal adjudication mechanisms are available, it is difficult to assess how often bargaining occurs in juvenile cases and under what circumstances.

### Knowing, intelligent, and voluntary pleas

Courts are required to ensure that all plea deals are entered knowingly, intelligently, and voluntarily. Notably, this inquiry is distinct from decisional competency (Grisso et al., 2003) and adjudicative competency. Rather, evaluating plea validity involves determining whether the specific decision to plead guilty (“no contest,” or in accordance with *Alford*) is made knowingly and intelligently (Redlich and Summers, 2012). Thus, before accepting a defendant’s plea, a judge must ensure that the defendant is not so impaired as to lack decisional capacity; that they understand the direct consequences of their plea; and that they were not promised anything (beyond the plea offer) in exchange for their plea (Redlich and Shteynberg, 2016).

The only published examination of existing state statutes regarding juvenile plea proceedings (now notably 30-years-old), observed that 10 states had no explicit regulations in place for guilty pleas entered in juvenile court, while statutes in another 40 states (and D.C.) exhibited vast variability in both the extent of their rules and the details provided (Sanborn, 1992). In juvenile courts, youths may enter the equivalent of a guilty plea informally, or without a written record. Unsurprisingly, juvenile defendants demonstrate a limited understanding of the plea process and the consequences of pleading guilty (Daftary-Kapur and Zottoli, 2014), and their deficits are greater than those exhibited by adult defendants (Redlich and Shteynberg, 2016; Zottoli and Daftary-Kapur, 2019). Further, this lack of adequate protections for juvenile defendants is not a uniquely American problem (Helm, 2021).

Even when youths are represented by counsel, that assistance seems to vary both quantitatively and qualitatively. Zottoli et al. (2016) found that juvenile defendants in New York reported fewer meetings, on average, with their attorneys relative to adult defendants. In known exonerations of youthful offenders, a higher percentage involved ineffective assistance of counsel than in exonerations of adults (Tepfer et al., 2009). Buffey’s case serves as an exemplar of these common problems. Moreover, while guardians can offer additional protections to juvenile defendants, their interests do not consistently align with the interests of their children, and they often show the same shortcomings in plea-related knowledge (Cavanagh and Cauffman, 2017; Fountain and Woolard, 2021).

Issues of voluntariness also abound within the juvenile system. While plea validity hinges on its’ voluntariness (which in turn, depends on the defendant’s ability to knowingly relinquish due process rights), courts have generally avoided all but the broadest definitions of voluntariness in juvenile cases (Cabell and Marsh, 2020). This resistance is rooted in the historical perception of the juvenile court as serving a *different purpose* than the criminal court. Justice Blackmun, writing for the majority in McKeiver v. Pennsylvania (1971; a case denying the right to jury trial for youths tried in juvenile courts) wrote, *“compelling a jury trial might remake the proceeding into a fully adversary process, and effectively end the idealistic prospect of an intimate, informal protective proceeding.”* Consequently, standards for knowing, intelligent, and voluntary pleas in juvenile cases have yet to be adequately defined.

### The plea colloquy

The process of validating a plea plays out during a plea colloquy: a brief hearing in which a plea is entered (Wilford et al., 2019). These hearings are notoriously monotonous, with defendants providing predictable, cursory, one-word answers to judges’ inquiries (Berube et al., 2022; Dezember et al., 2021). In some jurisdictions, defendants also receive a tender-of-plea form, which can serve to confirm plea validity. Thirty states have statewide tender-of-plea forms for adults, but only 17 have a juvenile equivalent (Redlich and Bonventre, 2015). Unfortunately, these forms are often written at reading levels that significantly exceed that of the average adult defendant; content analyses further reveal numerous gaps in the topics covered versus what criminal defendants should know about their plea convictions (Redlich and Bonventre, 2015). In one study, justice-involved youth in Massachusetts comprehended an average of 14% of the legal terminology routinely used in plea colloquies and forms (Kaban and Quinlan, 2004). Likewise, a systematic analysis of plea colloquies in California revealed that juvenile colloquies were significantly shorter than adult plea colloquies (7 vs. 13 minutes, respectively; Redlich et al., 2022). Another observational analysis reported juvenile plea colloquies lasted 5–10 minutes on average (Sanborn, 2002). While durations may not fully reflect the quality of these hearings, it is difficult to imagine that individual pleas (particularly youthful pleas) could be adequately assessed as knowing, intelligent, and voluntary in 10 minutes or fewer.

### Paths to reform: challenges and opportunities

Research has already demonstrated that juvenile defendants have less time with their attorneys (Zottoli et al., 2016), are subject to shorter plea colloquies (Redlich et al., 2022), are more vulnerable to perceived pressure from authority figures (including parents and attorneys; Fountain and Woolard, 2021; Malloy et al., 2014), and are more likely to plead guilty falsely (Helm et al., 2018). Yet much more research is required to illuminate how to protect youths facing criminal accusations.

Despite recent advances in this area, there is still far less research on juvenile plea decision-making relative to adult decision-making. There are many possible explanations for this asymmetry. First, field researchers in this area face many logistical barriers. Juvenile plea colloquies are typically closed to the public (Redlich et al., 2022), and juvenile court records are often sealed or unavailable for public review. Further, practices and levels of discretion in juvenile cases vary substantially across jurisdictions. Added judicial discretion and increased potential for informal disposition can lead to even greater variability in case outcomes, which in turn can make measurement and comparison across cases and jurisdictions difficult. Further, there are simply fewer juvenile plea dispositions relative to adult plea dispositions (evidenced by arrests of adults outnumbering those of youth by nearly 20 to 1 [in 2019]; Puzzanchera et al., 2022).

There are obstacles to experimental research as well. It is much more difficult to obtain ethical approval for studies involving youth (versus adult) participants (given their status as vulnerable participants). Further, youths’ study participation often requires parental/guardian consent, as well as participant assent. This added step increases the complexity of recruitment exponentially. Nonetheless, these efforts are particularly important given youths’ decisional vulnerabilities, coupled with the ubiquity of pleas within the legal system. Research is particularly critical in identifying effective strategies to improve youths’ understanding of the process and consequences of plea decisions, as well as discover meaningful safeguards against the pressures they face within this process.

## Actionable recommendations

Perhaps the most immediate question is *who* can act as a shield for youthful defendants? While researchers and legal scholars frequently cite the need for added protections of youth (as in the latest Scientific Review Paper on police-induced confessions; Kassin et al., 2024; Meek, 2019, for a legal scholar’s perspective), there is much less agreement regarding what form these protections should take. Most agree that youthful defendants should always be provided with legal representation, for all court proceedings. However, given the limited time and resources available to criminal defense attorneys, they may not be positioned to fully protect their youthful clients (Daftary-Kapur and Zottoli, 2014; Zottoli and Daftary-Kapur, 2019). Further, few attorneys are trained on effectively counseling youth, let alone using developmental or psychological science to effectively engage them throughout this complex process. Defenders also face the challenging ethical mandate to zealously represent their clients’ *expressed* interests (even if those interests are ill-conceived). In some indigent defender organizations, protective roles are assigned to Social Service Advocates–specially trained caseworkers tasked with supporting and guiding defendants through the legal process (Geurin et al., 2013; Phillippi et al., 2021). Yet many defense organizations lack the resources to hire these staff (Hollinger, 2020). Expanding these services requires significant budgetary, research, and training investments, which have been the focus of indigent defender advocacy for decades, with limited success (Hollinger, 2020).

Short of system-wide funding increases for indigent defense, policymakers and courts could also provide youthful defendants with a specialized advocate whose energy can be exclusively directed toward supporting them—perhaps like a guardian-ad-litem (GAL), with some noted differences. GALs are appointed by courts to protect the interests of individuals who cannot advocate for themselves (often minors in custody disputes). Yet the scope of GAL appointments may be expanded to protecting children’s interests as defendants. However, GALs are often empowered to make decisions that serve a child’s *best* interest (sometimes contrary to their *expressed wishes*); thus, restricting the GAL to advisory and supportive functions (without decision-making powers) would be critical in preserving youths’ rights in the plea context. Combining defense counsel with a GAL might simultaneously meet the need to advocate for youths’ legal interests, while also helping them comprehend their situations.

## Conclusion

Most courts at least attempt to provide interpreters for criminal defendants who speak limited English (Hale, 2020)—perhaps youth require a similar service. Young defendants, in many ways, speak a different language than adults (“lawyer dog” vs. “lawyer, dawg”). As such, they should be afforded an opportunity to have the language of the court translated in their own vernacular, and likewise, have *their language* translated appropriately to the Courts. Without added protections, and with limited research access to juvenile courts, we will remain blind to the knowingness, intelligence, and voluntariness of youthful (guilty) pleas.
